# The role of the renal afferent and efferent nerve fibers in heart failure

**DOI:** 10.3389/fphys.2015.00270

**Published:** 2015-10-01

**Authors:** Lindsea C. Booth, Clive N. May, Song T. Yao

**Affiliations:** Florey Institute of Neuroscience and Mental Health, University of MelbourneMelbourne, VIC, Australia

**Keywords:** radiofrequency-ablation, renal denervation, arterial baroreflex, renal sympathetic nerve activity, renal afferent

## Abstract

Renal nerves contain afferent, sensory and efferent, sympathetic nerve fibers. In heart failure (HF) there is an increase in renal sympathetic nerve activity (RSNA), which can lead to renal vasoconstriction, increased renin release and sodium retention. These changes are thought to contribute to renal dysfunction, which is predictive of poor outcome in patients with HF. In contrast, the role of the renal afferent nerves remains largely unexplored in HF. This is somewhat surprising as there are multiple triggers in HF that have the potential to increase afferent nerve activity, including increased venous pressure and reduced kidney perfusion. Some of the few studies investigating renal afferents in HF have suggested that at least the sympatho-inhibitory reno-renal reflex is blunted. In experimentally induced HF, renal denervation, both surgical and catheter-based, has been associated with some improvements in renal and cardiac function. It remains unknown whether the effects are due to removal of the efferent renal nerve fibers or afferent renal nerve fibers, or a combination of both. Here, we review the effects of HF on renal efferent and afferent nerve function and critically assess the latest evidence supporting renal denervation as a potential treatment in HF.

Patients with heart failure (HF) have a poor prognosis, with a 5-year mortality rate of 75% (Levy et al., 2002). In HF, the reduced cardiac output and inadequate perfusion of organs triggers a complex set of compensatory mechanisms, including activation of the sympathetic nervous system (SNS) and renin-angiotensin-aldosterone system (RAAS) (Weiss et al., 2003). The increased renal sympathetic nerve activity (RSNA) leads to increased release of renin, renal vasoconstriction (RVR), reduced renal blood flow (RBF), and renal sodium and water retention, with renal dysfunction being predictive of poor outcome (Goldberg et al., 2005; Petersson et al., 2005; Jose et al., 2006; Aspromonte et al., 2011). Although multiple therapies have been developed for the treatment of HF, these have only partially reduced the disease burden. As such, new treatments and novel approaches for tackling the disease are desperately needed.

Recently, catheter-based radiofrequency ablation of the renal nerves has been used as a treatment for drug-resistant hypertension and it has been proposed as a treatment for HF. The beneficial effects of renal denervation (RDN) are thought to depend on destruction of both the efferent, sympathetic and the afferent, sensory renal nerve fibers. This review will focus on the effects of the renal efferent and afferent nerve fibers in HF. We will also review the latest evidence supporting catheter-based RDN as a treatment in HF.

## Increased sympathetic nerve activity in heart failure

There are differential increases in sympathetic activity to individual organs in HF, as shown by measurement of regional noradrenaline spillover in HF patients (Hasking et al., 1986). It has been shown in HF patients and animal models of HF that the greatest increase in SNA is to the heart, with a smaller increase to the kidneys (Hasking et al., 1986; Ramchandra et al., 2009a). Importantly, these increases in SNA to the heart and kidneys are predictive of poor outcome (Kaye et al., 1995; Petersson et al., 2005). Relatively large increases in RSNA have been reported in rats 4 weeks after myocardial infarction, with burst incidence increased from 35 to 47% (DiBona et al., 1988; Feng et al., 1994), and RSNA was increased from 30 to 60% of maximum in rabbits with pacing-induced HF (Liu et al., 2000, 2001). However, such large increases in RSNA are not always seen in the early stages of HF. For example, in sheep paced into HF (ejection fractions: 35–40%), cardiac SNA (CSNA) was increased three-fold, whereas a modest increase in RSNA was only observed when activity was expressed as bursts per minute, mostly driven by an increase in heart rate (HR) (Ramchandra et al., 2009b). Similarly in patients, renal noradrenaline spillover is not increased in mild HF (ejection fraction: 29%) but is significantly increased in severe HF (ejection fraction: 18%) (Rundqvist et al., 1997).

## Causes of increased renal efferent sympathetic nerve activity in HF

**Figure 1:**
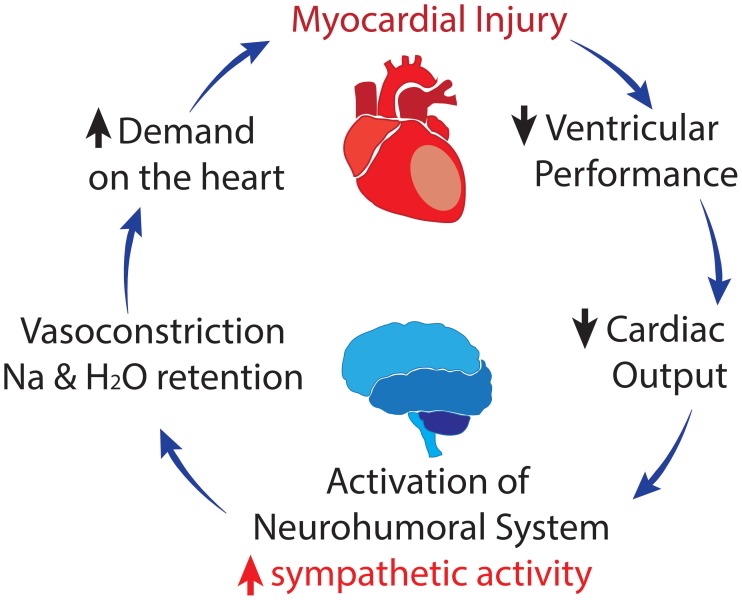
**Vicious cycle of heart failure; where activation of the sympathetic and renin-angiotensin-aldosterone systems contributes to a progressive deterioration in cardiac function**.

Increased sympathetic drive to the kidneys in HF causes renal vasoconstriction, RAAS activation and sodium and water retention, leading to increases in blood volume and BP. Although this may initially be beneficial in improving perfusion, with deteriorating heart function, the enhanced sympathetic drive puts extra load on an already stressed cardiovascular system. This leads to a vicious cycle of increasingly high levels of sympathetic drive and a progressively deteriorating cardiac system (Figure 1). The mechanisms underlying the specific increase in sympathetic drive to the heart and kidneys in HF remain incompletely understood.

### Blunted arterial baroreflex control in HF

There is extensive evidence indicating that altered control by inhibitory and excitatory reflexes contributes to the sympathoexcitation in HF. The arterial baroreflex is the main inhibitory reflex controlling SNA and desensitization of this reflex could contribute to increased SNA levels. Desensitized arterial baroreflex control has been shown for muscle SNA in HF patients (Leimbach et al., 1986; Grassi et al., 1995), and for RSNA in rabbits (Liu et al., 2000), dogs (Wang et al., 1991), and rats (Feng et al., 1994; DiBona and Sawin, 1995) with experimentally-induced HF. Impaired baroreflex control of SNA has, however, not been shown in all studies. For example, preserved arterial baroreflex control of muscle SNA has been reported in patients with HF (Dibner-Dunlap et al., 1996) and it has been argued that even in patients with advanced HF, the baroreflex control of muscle SNA is not desensitized (Floras, 2001). In anesthetized dogs with pacing-induced HF, baroreflex control of RSNA was preserved although baroreflex control of HR was desensitized (Dibner-Dunlap and Thames, 1989). Similarly, in ovine pacing-induced HF, the baroreflex control of RSNA and CSNA were unchanged; however, there was impaired baroreflex control of HR (Watson et al., 2007; Ramchandra et al., 2009a).

### Attenuated cardiopulmonary reflex inhibition of SNA in HF

The increase in blood volume and thus cardiac pressures that occur in HF would be expected to stimulate the cardiopulmonary reflex and inhibit SNA. There is extensive evidence that in HF the sensitivity of this inhibitory reflex is reduced. In rats, the reflex decrease in RSNA in response to acute volume expansion is reduced (DiBona et al., 1988). Similarly, we demonstrated that inhibition of RSNA, as well as CSNA, by volume expansion in normal sheep was largely abolished in sheep with HF (Ramchandra et al., 2009b). These findings indicate that the cardiopulmonary mechanoreceptor reflex is largely ineffective in HF, allowing SNA to remain elevated in the face of expanded blood volume.

### Exaggerated responses to chemoreceptor stimulation in HF

There is also evidence that the sympathoexcitatory chemoreflex is sensitized in patients with HF and that this is strongly associated with severity of the disease and poor outcome (Chua et al., 1997; Ponikowski et al., 2001). In support of these findings, in rabbits with pacing-induced HF, deactivation of the carotid chemoreflex with hyperoxia or cryoablation of the carotid bodies decreased RSNA (Sun et al., 1999; Marcus et al., 2014). Similarly, in ovine pacing-induced HF, deactivation of the carotid chemoreflex with hyperoxia decreased CSNA (Xing et al., 2014). Although, as described above, RSNA burst rate is not significantly elevated in this ovine HF model, hyperoxia decreased RSNA, as expressed as bursts/minute due to a decrease in HR (Xing et al., 2014). There is evidence that increased angiotensin II (AngII) levels, acting on angiotensin type-1 (AT-1) receptors in the carotid body, contributes to the sensitization of the chemoreflex in HF (Li et al., 2006).

### Central mechanisms stimulating SNA in HF

A more in-depth discussion of the central control of RSNA in HF is presented by Ramchandra et al. in this same special edition. As such we will only touch upon this briefly.

There is extensive evidence that the central angiotensinergic system plays a critical role in stimulating the increased SNA in HF. Blockade of central AT-1 receptors with losartan reduced the elevated RSNA in rats with HF induced by myocardial infarction (DiBona et al., 1995; Zhang et al., 1999) and reduced the high level of CSNA in ovine HF (Ramchandra et al., 2012). In addition, there are increased levels of AT1 receptors in a number of central autonomic areas, including the subfornical organ, paraventricular nucleus of the hypothalamus (PVN), nucleus of the solitary tract (NTS), and rostral ventrolateral medulla (RVLM) (Yoshimura et al., 2000; Gao et al., 2008). In particular, there is strong evidence that the PVN plays an important role in setting the increased levels of RSNA in HF (Patel, 2000), although the same is not true for CSNA (Ramchandra et al., 2013). In addition to AngII, a number of other factors within the PVN are likely to contribute to the changes in RSNA, including impaired nitric oxide function (Reddy et al., 2007), increased cytokine levels and oxidative stress (Guggilam et al., 2007; Kang et al., 2010).

## Renal afferent nerve fibers in HF

Compared with the widely studied renal efferents, there have been few studies of the renal afferent nerve fibers in HF. Renal afferent nerve activity is influenced by two main classes of receptors; mechanoreceptors and chemoreceptors. Mechanoreceptors are found within the renal parenchyma and in the wall of the renal pelvis (Niijima, 1975). These respond to increases in intra-renal pressure (Ueda et al., 1967) and can be stimulated experimentally by renal vein occlusion/compression in rats (Ueda et al., 1967), cats (Astrom and Crafoord, 1968), and dogs (Kostreva et al., 1981) and physical compression of the hilus of the kidney (Ueda et al., 1967; Astrom and Crafoord, 1968). Stimulation of renal mechanoreceptors with increases in renal venous pressure has been shown to lead to an increase in ipsilateral renal *afferent* activity and decreases in ipsilateral and contralateral *efferent* RSNA (Ueda et al., 1967; Kopp et al., 1985). Mirroring the decrease in contralateral efferent RSNA, mechanoreceptor activation generally results in decreased contralateral RVR (Kostreva et al., 1981). RVR on the ipsilateral side, however, has been reported to increase in direct response to increased renal venous pressure via non-neural mechanisms (Dilley et al., 1983; Kopp et al., 1985). Activation of renal mechanoreceptors has also been shown to affect renal function, with an increase in contralateral urine flow and contra- and ispilateral increases in sodium excretion (Kopp et al., 1985), although some studies have shown no change in ipsilateral sodium excretion and instead showed a decrease in potassium excretion (Dilley et al., 1983).

In addition to effects on the kidney, renal mechanoreceptor activation has been shown to inhibit SNA from the ansa subclavia and decrease right ventricular contractility and blood pressure (BP) (Kostreva et al., 1981). However, other studies have found no change in HR, BP or RBF with increases in intrarenal pressure (Kopp et al., 1984, 1985). A decrease in renal perfusion by balloon inflation in the aorta for 2 min (which is likely to activate chemo- and inhibit mechanoreceptors) caused an increase in hindlimb vascular resistance in anesthetized rabbits (Rankin et al., 1992). The decreased renal perfusion is thought to elicit hypoxic-driven release of local mediators, such prostaglandin E2, bradykinin, and adenosine, which stimulate renal afferents leading to neutrally-mediated increases in hindlimb vascular resistance (Ashton et al., 1994). The main responses to renal mechanoreceptor activation are abolished by spinal cord transection at T6, indicating that the mechanoreceptor reno-renal reflex is dependent on central integration (Francisco et al., 1980; Kopp et al., 1985).

The second class of renal sensory receptors are the chemoreceptors: R1 and R2 receptors, which are activated by the chemical environment of intrarenal tissue and renal pelvis, respectively (Recordati et al., 1978, 1980). R1 receptors are activated by renal ischaemia, stimulated experimentally by prolonged arterial and venous occlusion and systemic asphyxia (Recordati et al., 1978). R1 activation, induced by renal artery occlusion, is associated with an increase in ipsilateral efferent RSNA, which persists after spinal cord transection at T6 in rats (Recordati et al., 1982). R2 receptors are activated experimentally by backflow of concentrated urine (Rogenes, 1982), hypertonic NaCl, and hypotonic KCl (Recordati et al., 1980). Activation of R2 chemoreceptors results in an increase in both ipsilateral and contralateral efferent RSNA, which is more pronounced if backflow of urine is bilateral, and is variably accompanied by small increases in BP and HR (Recordati et al., 1982; Rogenes, 1982). Like the response of R1 receptors, the R2 response remains after spinal cord transection at T6 (Recordati et al., 1982) and is enhanced by transection at C3 (Rogenes, 1982); therefore, a reflex integrated at a spinal level.

Renal afferent nerve fibers are mainly unmyelinated (primarily C- fibers) with a small population of faster conducting, A-delta, myelinated fibers (Knuepfer and Schramm, 1987). Studies in rats indicate that the renal afferent nerve fibers project from the kidney to the ipsilateral dorsal root ganglia, between T6 and L2 (Donovan et al., 1983; Knuepfer and Schramm, 1987), with the peak number at T12–13. By stimulating myelinated renal afferent fibers, investigators have shown that there are direct projections from the kidney to the most medial segment of the nucleus gracilis and the caudal half of the NTS (Simon and Schramm, 1984) and fluorescent tracer studies between the kidneys and posterior medulla show that monosynaptic connections make up approximately 8% of renal afferents (Wyss and Donovan, 1984). In addition to these brainstem regions, in cats, electrical stimulation of the renal afferents effects activity of medullary neurons in the lateral tegmental field, paramedical reticular nucleus and dorsal vagal complex, and hypothalamic neurons in the lateral preoptic area, lateral hypothalamic area, and PVN (Calaresu and Ciriello, 1981). Additionally, the ventral medulla has been shown to receive input from renal afferents in the cat (Vizzard et al., 1992). Indeed, Xu et al. (2015) have recently shown that there is a neural connection from the RVLM to the PVN that is activated by stimulation of renal afferents. Importantly for the role of the renal afferents in HF, the same authors have previously shown that RVLM projecting PVN neurons are more active in rats with chronic HF (Xu et al., 2012). As the RVLM plays a crucial role in the regulation of SNA, this may be a pathway by which renal afferent activation in HF influences sympathetic tone; however, this remains to be confirmed. Electrical stimulation of renal afferent nerve fibers has also been studied using Fos (a marker of neuronal activation) immunohistochemistry (see Solano-Flores et al., 1997).

### Potential factors driving the changes in renal afferent activity in HF

There are very few studies that have examined the role of the renal afferent nerve fibers in HF. HF is associated with a number of symptoms which would be expected to stimulate renal afferent activity, such increased venous pressure and decreased RBF. Kopp et al. showed that the inhibitory mechanoreceptor reno-renal reflex is blunted in HF, due to high circulating AngII (Kopp et al., 2003) and activation of endothelin A receptors (Kopp et al., 2010). Blunting of the inhibitory reno-renal reflex may be a mechanism by which sodium is retained and efferent sympathetic drive to non-renal vascular beds is stimulated in HF. It is unknown whether the excitatory renal-chemoreflex is enhanced in HF, potentially in parallel with the enhanced arterial chemoreflex.

## Ablation of the renal nerve fibers in heart failure: evidence for potential benefit following catheter-based RDN

Discussed above are some of the potential factors stimulating SNA in HF and the effects of the renal sympathetic and sensory nerves. The critical question is whether removing the effect of these nerves is beneficial in HF. The development of catheter-based renal nerve ablation has led to increasing interest in RDN as a treatment for hypertension and HF. Although not without controversy (Bhatt et al., 2014), RDN has been shown to be effective in lowering BP in patients with drug-resistant hypertension (Krum et al., 2009; Esler et al., 2010). Recently, the First Report of the Global SYMPLICITY Registry showed a significant reduction in 24 h ambulatory BP after RDN in nearly 1000 patients (Böhm et al., 2015), supporting the population effect of catheter-RDN.

**Figure 2:**
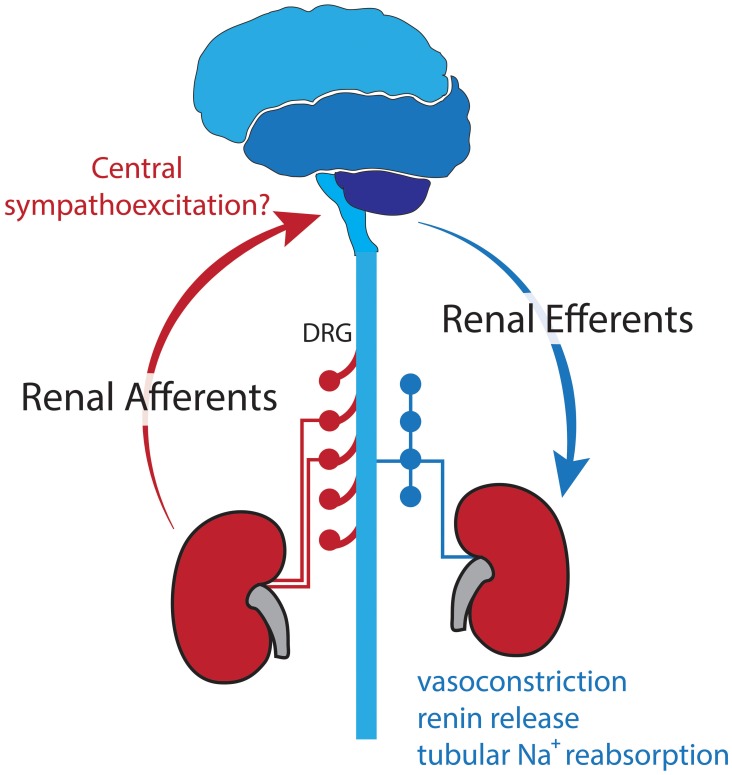
**Sympathetic efferent and sensory afferent renal nerves postulated to be interrupted with RDN**.

The BP lowering effects of RDN are *postulated* to be due to destruction of both renal efferent and afferent nerve fibers (Figure 2). As outlined previously, efferent renal nerves play a major role in stimulating renin release, renal vasoconstriction, and sodium retention (DiBona and Kopp, 1997), thus removal of these nerves decreases BP. It has also been suggested that in hypertension, increased afferent renal nerve activity causes a reflex increase in sympathetic outflow and worsening hypertension (Katholi and Woods, 1987; Campese et al., 1995) and there is evidence that ablation of the afferent nerve fibers reduced muscle SNA (Schlaich et al., 2009) and plasma noradrenaline (Ezzahti et al., 2014). These effects of both efferent and afferent RDN are likely to be beneficial in HF.

Successful destruction of the renal nerves depends heavily on the ablation sites in relation to the renal nerves. It has recently been highlighted that although the number of renal nerves is higher in proximal regions of the renal artery, the renal nerves are closest to the renal artery in distal regions in humans (Sakakura et al., 2014), pigs (Tellez et al., 2013), and sheep (Booth et al., 2015a). Therefore, starting ablations as close as possible to the kidney may be the most effective method of ablating the renal nerves. We have previously shown a ~80% reduction in renal noradrenaline levels with six ablations started as close as anatomically possible to the kidney in sheep (Booth et al., 2015b).

### Clinical studies of RDN in HF

While it is intuitive to use RDN in hypertensive patients to reduce BP, this is less so in HF where BP is reduced in the majority of cases. However, the ability of RDN to reduce RSNA and thus the increased renal vasoconstriction, renin release, and sodium retention is likely to have beneficial effects. Indeed, a safety trial in HF patients showed that there were no significant reductions in BP following RDN in the seven systolic HF patients and, importantly, RDN was associated with an increase in 6-min walk distance 6 months after RDN (Davies et al., 2013). Importantly, in a pilot study RDN was shown to reduce ventricular tachyarrhythmias in two patients with chronic HF (Ukena et al., 2012). Further, RDN trials in hypertensive patients with cardiomyopathy have shown that 6 months after RDN patients had reduced left ventricular mass (Doltra et al., 2014; Mahfoud et al., 2014) and increased EF (Mahfoud et al., 2014). Larger clinical trials of renal denervation in HF are ongoing (Verloop et al., 2013).

### Effect of RDN on renal function in experimental HF

As previously described, RSNA is increased in severe HF and this has detrimental actions suggesting that RDN would be beneficial. Indeed, bilateral surgical RDN attenuated the sodium retention following myocardial infarction in rats (DiBona and Sawin, 1991; Souza et al., 2004) and in dogs with HF (Villarreal et al., 1994). Studies in rats, 3–4 weeks after myocardial infarction (LVEDP ~ 18 mmHg), showed impaired water and sodium excretion following an acute salt load, a finding reversed by prior RDN (DiBona et al., 1988). Increased sodium reabsorption in HF is likely to be at least partially driven by increased expression of the Na-K-2Cl cotransporter in the thick ascending loop of Henle, which has been shown in HF rats and was reduced following RDN (Torp et al., 2012). In addition to altered sodium handling, large myocardial infarcts have been associated with increased RVR, decreased renal plasma flow and an inability to increase glomerular filtration rate after volume loading (Hostetter et al., 1983). As mentioned above, reduced RBF and renal dysfunction are predictive of poor outcome in HF patients (Goldberg et al., 2005; Petersson et al., 2005; Jose et al., 2006). In rabbits paced into HF, unilateral RDN prevented the reduction in RBF, increase in RVR and upregulation of AT-1 receptor expression in renal cortical blood vessels otherwise seen with HF (Clayton et al., 2011). Together these studies indicate that RDN improves renal function in experimental models of HF, probably mainly by efferent denervation.

### Effect of RDN on cardiac function in experimental HF

Surgical RDN has been shown to reduce left ventricular filling pressure and improve function following myocardial infarction in rats (Nozawa et al., 2002; Hu et al., 2014a); while, catheter-based RDN, prior to pacing-induced HF, has been shown to reduce the incidence of atrial and ventricular fibrillation and left ventricular filling pressure in dogs (Zhao et al., 2013; Guo et al., 2014). In contrast, in rabbits with pacing–induced HF, unilateral surgical RDN did not improve cardiac function but reduced the sensitivity of the HR baroreflex and decreased plasma noradrenaline levels (Schiller et al., 2013). The majority of studies investigating RDN in HF have assessed the effects before or at the induction of HF. One of the few studies investigating RDN in established HF showed improved cardiac and renal function in rats when surgical denervation was performed 1 and 4 weeks post-myocardial infarction (Hu et al., 2014b). In addition, a recent study investigating the effects of surgical RDN in rats with cardiac dysfunction secondary to chronic pressure overload showed that RDN reduced myocardial fibrosis, increased cardiac β-adrenergic receptor expression and decreased cardiac AT-1 receptor levels (Li et al., 2015). In pacing-induced ovine HF, the high resting level of CSNA was not reduced shortly after catheter-based RDN, but the baroreflex-mediated increase in CSNA in response to the fall in BP was inhibited following the procedure (Booth et al., 2015c). This lack of a reflex increase in CSNA resulted from a leftward shift of the CSNA arterial baroreflex curve (Booth et al., 2015c). These findings indicate that RDN can have beneficial cardiac effects in experimental HF, but further studies are required to determine the mechanisms involved. In addition, the extent to which any effects of RDN in HF depend on ablation of the afferent versus efferent nerve fibers remains, at present, unknown. This could be addressed using methods of selective denervation; such as destruction of renal afferent fibers with capsaicin (Foss et al., 2015) or destruction of renal efferent fibers with 6-hydroxydopamine (LeNoble et al., 1985).

## Conclusions

The renal nerves are made up of afferent sensory and efferent sympathetic nerve fibers. Although the activities of both types of nerve fibers are postulated to increase in HF, the role of the sympathetic nerves have been much more widely investigated. In HF there is an increase in sympathetic outflow, especially to the heart and kidneys, which is associated with poor outcome. In experimentally induced HF, RDN, both surgical and catheter-based, has been associated with some improvements in renal and cardiac function. In contrast, the role of renal afferents remains largely unexplored in HF, although there are multiple triggers that could potentially increase afferent nerve activity. Some of the few studies investigating this have suggested that at least the inhibitory reno-renal reflex is blunted in HF. This may be one mechanism stimulating efferent sympathetic drive in HF, which leads to renal vasoconstriction, renin release, and sodium retention. Although the evidence outlined above indicates the beneficial effects of removing the renal nerves in HF, it remains unknown whether the effects are due to removal of the efferent, sympathetic renal nerves or sensory, afferent renal nerves, or a combination of both.

## Sources of funding

This work was supported by National Health and Medical Research Council of Australia (NHMRC) and the Victorian Government's Operational Infrastructure Support Program. LB is supported by a NHMRC Early Career Fellowship and CM was supported by a NHMRC Research Fellowship.

### Conflict of interest statement

Clive N. May has received honoraria and travel support for presentations from Medtronic. The authors declare that the research was conducted in the absence of any commercial or financial relationships that could be construed as a potential conflict of interest.
